# Evaluation of the orally administered calcium alginate aerogel on the changes of gut microbiota and hepatic and renal function of Wistar rats

**DOI:** 10.1371/journal.pone.0247633

**Published:** 2021-04-28

**Authors:** Mohammad A. A. Al-Najjar, Tamara Athamneh, Reem AbuTayeh, Iman Basheti, Claudia Leopold, Pavel Gurikov, Irina Smirnova

**Affiliations:** 1 Department of Pharmaceutical Sciences and Pharmaceutics, Faculty of Pharmacy Applied Science Private University, Amman, Jordan; 2 Institute of Thermal Separation Processes, Hamburg University of Technology, Hamburg, Germany; 3 Institute of Pharmacy, University of Hamburg, Hamburg, Germany; 4 Laboratory for Development and Modelling of Novel Nanoporous Materials, Hamburg University of Technology, Hamburg, Germany; North-Eastern Hill University, INDIA

## Abstract

The present study evaluates the effect of calcium alginate aerogel as a potential drug carrier, on the liver and kidney functions, and on the gut microbiota of Wistar rats. The studied alginate aerogel was prepared in the form of nanoparticles using the jet cutting technique, and they were characterized in terms of specific surface areas, outer morphology and particle size distribution. For the *in vivo* study, calcium alginate aerogel was administered orally, and liver and kidney functions were tested for one week and for four weeks in two distinct studies. During the short-term *in vivo* study, feces samples were collected for bacterial DNA extraction followed by 16S rRNA gene sequencing analyses to detect changes in gut microbiota. Results showed that the prepared alginate aerogel has an average BET-specific surface area of around 540 m^2^/g, with a pore volume of 7.4 cc/g, and pore width of 30–50 nm. The *in vivo* study revealed that the levels of the studied kidney and liver enzymes didn’t exceed the highest level of the normal range. The study of gut microbiota showed different patterns; certain groups of bacteria, such as Clostridia and Bacteriodia, increased during the aerogels regime and continued to increase after the aerogel was stopped. While other groups such as *Erysipelotrichia*, and *Candidatus saccharibacteria* increased during aerogels treatment, and then decreased again after one month. Members of the Bacilli class showed a unique trend, that is, after being the most abundant group (63%) at time 0, their relative abundance decreased dramatically until it reached < 5%; which was the case even after stopping the aerogel treatment.

## Introduction

Aerogels terminology was emerged for the first time in 1931 by Samuel Kistler, who defined them as the materials retaining their pore and network structure intact upon exchanging their pore liquid with gas [1]. After 60 years of their discovery, organic aerogels were proposed for biomedical and drug delivery applications due to their large inner surface area and open pore structure [2]. The outstanding features of this material class allows not just to increase the loading efficiency, but also to improve the bioavailability, stability and the release kinetics of the loaded molecules.

Research on aerogels for the oral drug administration initially focused on the use of silica aerogels because of its flexible and well-known sol-gel chemistry, as well as the possibilities of derivatization [3]. Nevertheless, the use of silica aerogels in pharmaceutical formulations designed for oral administration still poses certain concerns because of their limited biodegradability. Therefore, a further need arises to develop an aerogel carrier that is both biocompatible and biodegradable. This may be possible by using natural organic materials or biodegradable polymers, such as alginate [4] hyaluronic acid [5], chitosan, cellulose [6] and proteins [7].

Alginate (Alg), extracted from brown algae, is a linear copolymer composed of ß-1,4-D-mannuronic acid and α-L-guluronic acid monomers, which are interspersed homogenous or heterogeneous block-like patterns. Alginates have been used in the food and pharmaceutics industry because it is non-toxic, inexpensive, biodegradable and biocompatible [8, 9]. Polyvalent cations, such as Ca^2+^, Ba^2+^ or Sr^2+^, can induce ionic cross-linking of Alg, transforming it from a soluble to an insoluble (gel) form.

Alg has been widely used in food products and as a pharmaceutical additive, such as a gelling agent and a tablet disintegrant [10, 11]. It has also been used in biomedical applications, including drug delivery and tissue engineering [12]. Like other edible dietary fibers, Alg and its oligomer derivatives are resistant to digestion by human endogenous enzymes. However, it can be digested significantly by human gut microbiota [13]. Despite its importance, little is known about gut microbiota which is responsible for the degradation of Alg.

The European Food Safety Authority (EFSA) has declared the safety of alginic acid and its salts, as well as their fermentation products. *In vivo* tests for the absorption and excretion of aqueous solutions and suspensions of alginic acid and its salts in animals showed their inability to be absorbed or metabolized by enzymes present in the gastrointestinal tract regardless of the form used during administration. However, they would be partially degraded by fermentation during their passage through the large intestine by the action of the anaerobic intestinal microbiota, causing caecal enlargement which was considered by the EFSA as an adaptive process related to the high doses tested as food additives [14]. Nevertheless, no previous *in vivo* study has examined the effect of calcium alginate (Ca-Alg) in the *form* of aerogel on the liver, kidney or the bacterial community in the intestines.

Gut microbiota are essential components of the human digestive system, as they help in the degradation of the ingested food, especially those for which the human body does not have the required enzymes [15, 16]. Gut microbiota can also inhibit the blooming of pathogenic microorganisms by competing with them on the nutrients’ resources, or by producing chemical substances to inhibit their growth. However, it has been shown that the degradation of the ingested digestible fibers, such as Alg, occurs mainly by fermented bacteria in the colon [17]. The products of this process are short-chain fatty acids (SCFA), which are beneficial for colon inflammation and act as anticancer. Brownlee et al. reported that the incubation of Alg and human fecal microflora demonstrated that most of Alg degradation is accompanied by a change in the gut microbiota. Also, such incubation leads to the production of SCFA and gas after 24 hours [17].

The goal of this study is to assess the safety of Ca-Alg aerogel through studying its effect on the kidney and liver, as it has been proposed widely in the literature for drug delivery applications. Besides, as Alg is degraded in the intestinal by the gut microbiota, the effect of Ca-Alg aerogel on the intestinal microbial community will also be evaluated.

## Materials and methods

### Materials

Alginic acid sodium salt was supplied by BASF, Germany. Calcium chloride (CaCl_2_) was purchased from Th. Geyer GmbH & Co. KG, Germany. Carbon dioxide (purity ≥ 99.5%) was supplied by Praxair (Germany), ethanol 99.8% was obtained from Carl Roth (Germany). All chemicals were used as received. Commercial diagnostic kits were used to measure ALP, and creatinine kits were purchased from Biostsystems S.A, Spain. The MO BIO’S PowerMax Soil DNA Isolation Kit were purchased from MO BIO Laboratories, USA. Deionized water was used throughout the study.

### Methods

#### Preparation of aerogel particles

The first step in the preparation of the aerogel particles was to prepare the stock solution; for that sodium alginate powder (Alg) was added to distilled water in a concentration of 1% w/w and was kept under magnetic stirring overnight at room temperature. To prepare the hydrogel particles, the approach described by Preibisch et al. [18] was followed, using Jet Cutter Type S from geniaLab, Braunschweig (Germany). In the jet-cutting process, the alginate solution was ejected through a nozzle (350 μm diameter) by compressed air (1–3 bar) to form a jet (mass flow rate of 1 g/s). The jet was cut in separate particles with a rotating disc (40 wires with a 100 μm of wire diameter of cutting discs, cylinder ratio of 5). After passing through the disc, the polymer solution fell downwards into the gelation bath with CaCl_2_ solution of 5 g/L. To avoid agglomeration of the gel particles, the volume of the gelation bath was at least four times the total volume of the processed biopolymer solution, and the content of the baths was stirred with a magnetic bar (60 rpm). The separation distance between the nozzle and the gelation bath was kept at 50–70 cm. After collecting the gel particles, the stirring was continued for 60 min to ensure complete gelation and to avoid agglomeration. Gelled particles were then separated from the gelation bath via filtering and were proceeded for the solvent exchange.

Solvent exchange (water to ethanol) was performed on the collected particles stepwise (30, 60, 90, 100% v/v ethanol). To ensure complete exchange, the density of the soaking liquid after the last solvent exchange step was measured using a density meter (DMA4500, Anton Paar Company, Austria). The density was then recalculated into ethanol content. Resulting alcogel particles were eventually packed into a filter paper, and dried with supercritical CO_2_ in an autoclave at a constant temperature of 50°C and a pressure of 120 bar. Continuous flow of CO_2_ (20–80 g/min) was set until complete extraction of ethanol was done (2–4 hours). Afterwards, slow depressurization of the autoclave (1–3 bar/min) was performed. Once the ambient pressure was reached, the autoclave was opened and aerogel microspheres of calcium alginate (denoted as Ca-Alg hereafter) were collected and stored in well-sealed containers under dry conditions.

### Characterization of aerogel particles

Specific surface areas of aerogel particles were measured via low temperature nitrogen adsorption/desorption (BET) (Quantachrome Nova 3000e, Odelzhausen, Germany and Micromeritics TriStar II 3020, Germany). Prior to measurements, samples were dried for 20 h under vacuum (< 1 mPa) at 60°C.

Investigation of the outer morphology of the prepared aerogel particles was done via a scanning electron microscope (SEM) analysis (Zeiss Supra VP55, Jena, Germany) at an acceleration voltage of 3 kV and a working distance of 9.0–9.4 mm. Samples were gold-sputtered (10 nm thickness) prior to imaging in order to minimize charging and improve the image contrast.

Particle size distribution measurements were performed three times for each sample consisting of approximately 10,000 particles. Dynamic image analysis was performed using a Camsizer XT with X-Jet module (Retsch GmbH, Germany) with air pressurized at 50 kPa.

### Hepatic and renal function, and gut microbiota studies

A pilot study was done on a small number of animals divided into different groups to cover four dose levels of treatment substances. It was conducted for a short duration of time (1 week) in order to sight the maximum dose possible of Ca-Alg aerogels that has no major effects on renal or hepatic functions, and that does not demonstrate signs of morbidity or mortality. Doses were thoughtfully selected considering the reported oral LD_50_ of alginate (more than 5,000 mg/kg body weight) [19]. Yet the maximum feasible dose reached with minimal animal agitation was 2,000mg/Kg body weight.

In the second part of the study, the animals were exposed to a fixed dose level for two weeks. Regarding age and weight variations and husbandry conditions during this study, the Organization for Economic Cooperation and Development (OECD) guidelines were followed (code No. 206). All experimental protocols were approved by the Ethical Committee (IRB, Approval number: 2019-PHA-12, attached in S1 File) of Applied Science Private University (ASU), Jordan. All staff members in the animal house at the ASU are well trained to monitor any changes that might occur to the animal as a consequence of the intervention. Specifically, they monitor parameters such as weight changes, abnormal behaviors, ruffled fur, reduced mobility, body posture, or expression of specific body fluid markers. In case of any serious abnormal changes on the animal status, they are allowed to use the humane end-point protocol that it is approved by the Ethical committee at ASU. At the end of the experiments, rats were euthanized by exsanguination under general anesthesia using 2.5% thiopental sodium (Abbott Laboratories, North Point, Hong Kong) at 20 mg/kg intravenously.

### Animals

In both short term and long term *in vivo* studies, ten-week old, healthy Wistar Rats with an average weight of 240 ± 37 g were housed at a temperature of 21–23 °C and humidity of 35%–70% in controlled rooms, with 12 h light– 12 h dark cycles. Rats were identified and placed individually in clear-sided cages for ease of observation without disturbing their behavior. Wood shavings were used as bedding. Rats were fed a commercially available diet (Local Supplier, Jordan) and fresh water was freely offered.

The number of rats per group in the different experiments was selected according to the previously published work (examples; [20–22]), and depending on the strict regulations about “animal care and use” nationally and internationally, which stress on minimizing the number of animals used in research studies. These include but not limited to, “Animal Care and Use Program” at the University of California, Berkeley (https://acuc.berkeley.edu/about.html), and the Norwegian guideline for animal use (https://www.forskningsetikk.no/en/guidelines/science-and-technology/ethical-guidelines-for-the-use-of-animals-in-research/).

### Pilot *in vivo* study (7 days repeated doses)

A repeated oral dose for a one-week duration was performed to define the maximum possible dose of aerogels demonstrating safety in Wistar rats. Ca-Alg aerogel was administered orally at four dose levels of 50, 100, 250, and 500 mg in corresponding to an average dose of 200, 400, 1000, and 2000 mg/Kg (weightbody weight), respectively. The results were compared with two positive control groups that were given 50 and 500 mg of pristine sodium alginate (Na-Alg) corresponding to the average dose levels of 200 and 2000 mg/Kg body weight, respectively. Eventually, all the previous groups of Ca-Alg aerogel and Na-Alg treatments were referenced to a control group (placebo). The latter group received phosphate buffer in a dose equivalent to the maximum dose when it is administered as a vehicle system for the test substances. The control and treatment groups for the pilot study consisted of 4 rats/group (two females and two males/group). The solid powders of Ca-Alg aerogel and Na-Alg were respectively suspended in phosphate buffer (pH 7.4) and mixed well directly before the oral administration, because the rats refused to eat them in the dry state. For all groups, administration to rats was initiated at the same time everyday using an oral gavage.

### Long-term *in vivo* exposure study (15 days repeated dose study)

Based on the results from the previous section, the dose of 250 mg of Ca-Alg aerogel was selected to be further evaluated for the long term (15 days) exposure study Wistar rats were observed for any immediate or delayed renal and/or hepatic adverse effects that might occur when exposed to Ca-Alg aerogel or when exposed to Na-Alg respectively. of. For each tested substance, three animal groups were used that included a control, treatment and a satellite group. The control group consisted of 6 Wistar rats/group (3 males and 3 females), while the treatment and satellite groups consisted of 10 Wistar rats/group (5 males and 5 females). The Wistar rats in the treatment groups received the daily dosage of 250 mg of the test substances (Ca-Alg aerogel or Na-Alg) for 15 days, and they were sacrificed on day 16. While the satellite groups were treated for the same duration as that for the treatment groups, but were rather kept under observation for an additional 15 days post treatment, and they were sacrificed on day 30.

It is worth to mention that it was not possible to conduct the two studies of Ca-Alg aerogel and Na-Alg in parallel. For this reason, it was not convenient to use one control group for both. These control groups received oral phosphate buffer for 15 days only, and were subsequently monitored for 14 days further. Being a short-term experiment, the solid powders of Ca-Alg aerogel and Na-Alg were also suspended in a phosphate buffer (pH 7.4) and mixed well directly before the oral administration. For all groups, the administration to rats was initiated at the same time everyday using an oral gavage.

### Clinical and behavioral observation

In accordance with the OECD guidelines 407, animals were monitored daily for any abnormal clinical signs or changes in the behavior for the duration of the study period, with special attention at the first four hours after the administration of the test substance. Animals in the satellite and control groups were further observed for an additional 15 days without treatment administration. The satellite group allows the detection of any late-occurring clinical signs indicative of hepatic and/or renal impact through measuring Alkaline Phosphatase (ALP) and creatinine levels.

### Serum biochemistry and feces analyses

In the short-term sighting study, blood samples were collected every second day to measure levels of ALP and creatinine, using commercial diagnostic kits from Biostsystems S.A, Spain. The blood samples were collected from orbital sinus veins of the Wistar rats, which were then used to split-up the serum. This collected serum from the samples were then transferred into sterile Eppendorf tubes and stored in the deep freeze for further testing of biochemical parameters. Feces samples were also collected every second day to investigate the change in microbial community structure. The feces were gathered in a clean sterile 15 ml-screw -capped tubes, and were directly kept at -20°C until all the samples were collected. For the 15-days repeated dose study, only blood samples were collected on a weekly basis to measure the levels of Alkaline Phosphatase (ALP) and creatinine, that is on day 0, day 7, day 14, day 21 and day 30 (day 21 and day 30 applied for the control and satellite groups only).

### Intestinal microbial community analysis

DNA extraction from the collected feces samples was performed using MO BIO’S PowerMax Soil DNA Isolation Kit (MO BIO Laboratories, USA), following the manufacturer instruction. Polymerase Chain Reaction (PCR) purification, and sequencing of genomic DNA (gDNA) were then conducted using Mr. DNA Lab (Molecular Research LP, USA). The PCR amplification of the 16S rRNA gene and its subsequent sequencing were done using Illumina. The 16S rRNA gene V4 variable region PCR primers ill27F mod (AGRGTTTGATCMTGGCTCAG)/ ill519R and mod (GTNTTACNGCGGCKGCTG) with a barcode on the forward primer were used in 30 cycles using the HotStarTaq Plus Master Mix Kit (Qiagen, USA). After amplification, PCR products were checked in 2% agarose gel to determine the success of amplification and the relative intensity of bands.

Then the pooled and purified PCR product were used to prepare Illumina DNA library. Sequencing was performed with MR DNA on a microbiome sequencing (MiSeq) following the manufacturer’s guidelines. Sequenced data were processed using MR DNA analysis pipeline. In brief, sequences were joined, then were depleted of barcodes, after that, sequences < 150 bp as well as sequences with ambiguous base calls were removed. Sequences were denoised and operational taxonomic units (OUTs) were generated and chimeras removed. OTUs were defined by clustering at 3% divergence (97% similarity). Final OTUs were taxonomically classified using BLASTn against a curated database derived from RDP-II and NCBI. A package of different statistical analyses included in “R” software was used for hierarchical clustering (version 3.5.1, vegan Package).

## Results and discussion

### Aerogel particles characterization

The specific surface area and pore size are considered the major factors affecting the loading efficiency of the active pharmaceutical ingredient inside the aerogel. Higher surface area and smaller pore size usually result in a higher drug loading. The results of this study are comparable with the literature, showing an average BET specific surface area of around 540 m^2^/g, with a pore volume of 7.4 cm^3^/g, and pore width of 30–50 nm. High surface areas and mesoporosity for supercritically dried Ca-Alg aerogels have been reported by various researchers [4, 5, 23].

**Fig 1:**
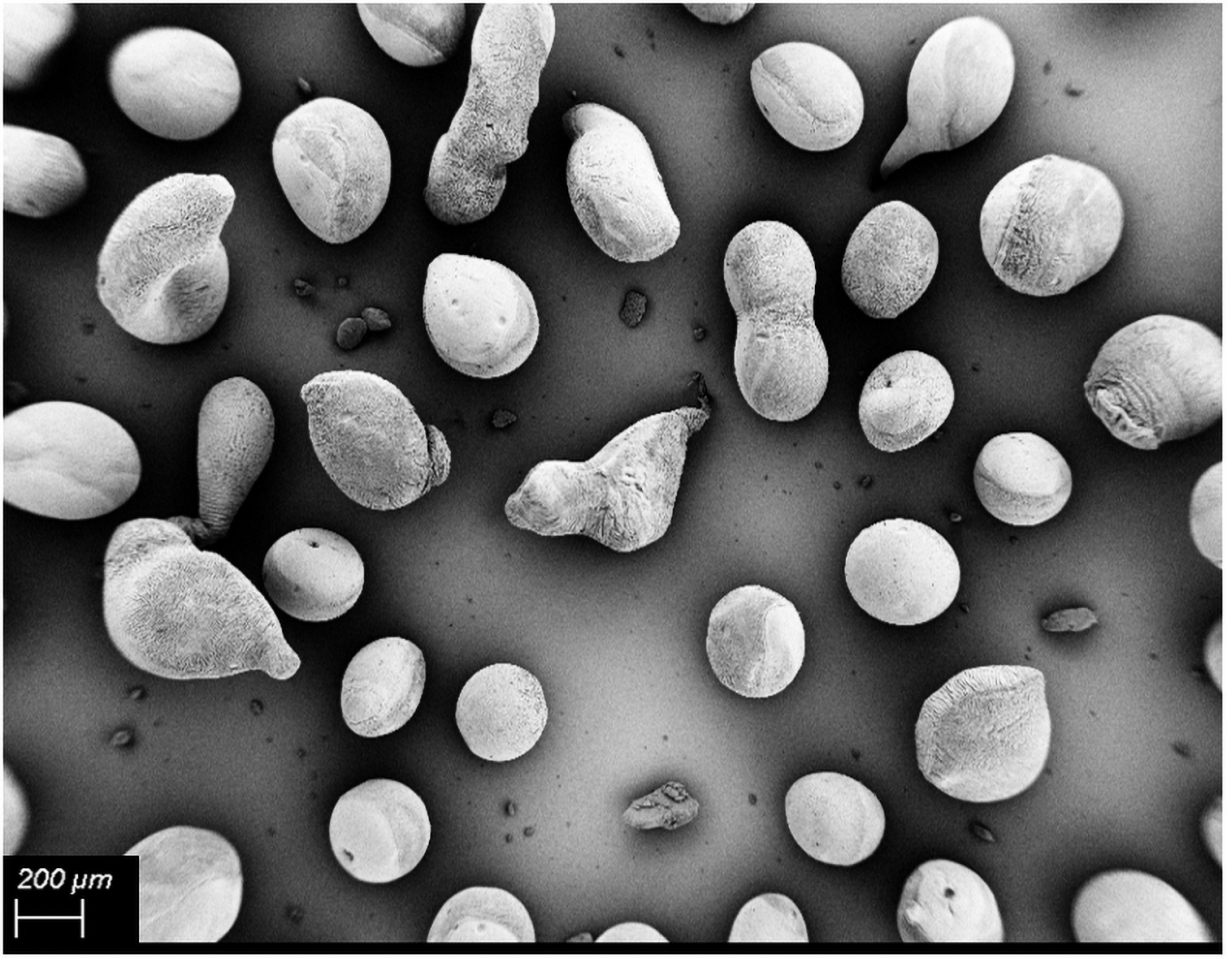
Scanning electron micrographs of Alg aerogel particles which were prepared by a jet cutting process with a subsequent drying step by supercritical CO_2_.

The SEM micrographs of the external morphology of the tested aerogel particles are shown in Fig 1. The majority of particles are spherical, with some elongated or tailed particles. Such deformation of particles when using the jet cutter technique was observed by Preibisch et al. [18] and was referred to the high cylinder ratio (a cylinder ratio of 5, which means gel cylinders were 5 times higher than their diameter).

### Hepatic and renal function *in vivo* study

#### Pilot *in vivo* study (7 days repeated doses)

**Fig 2:**
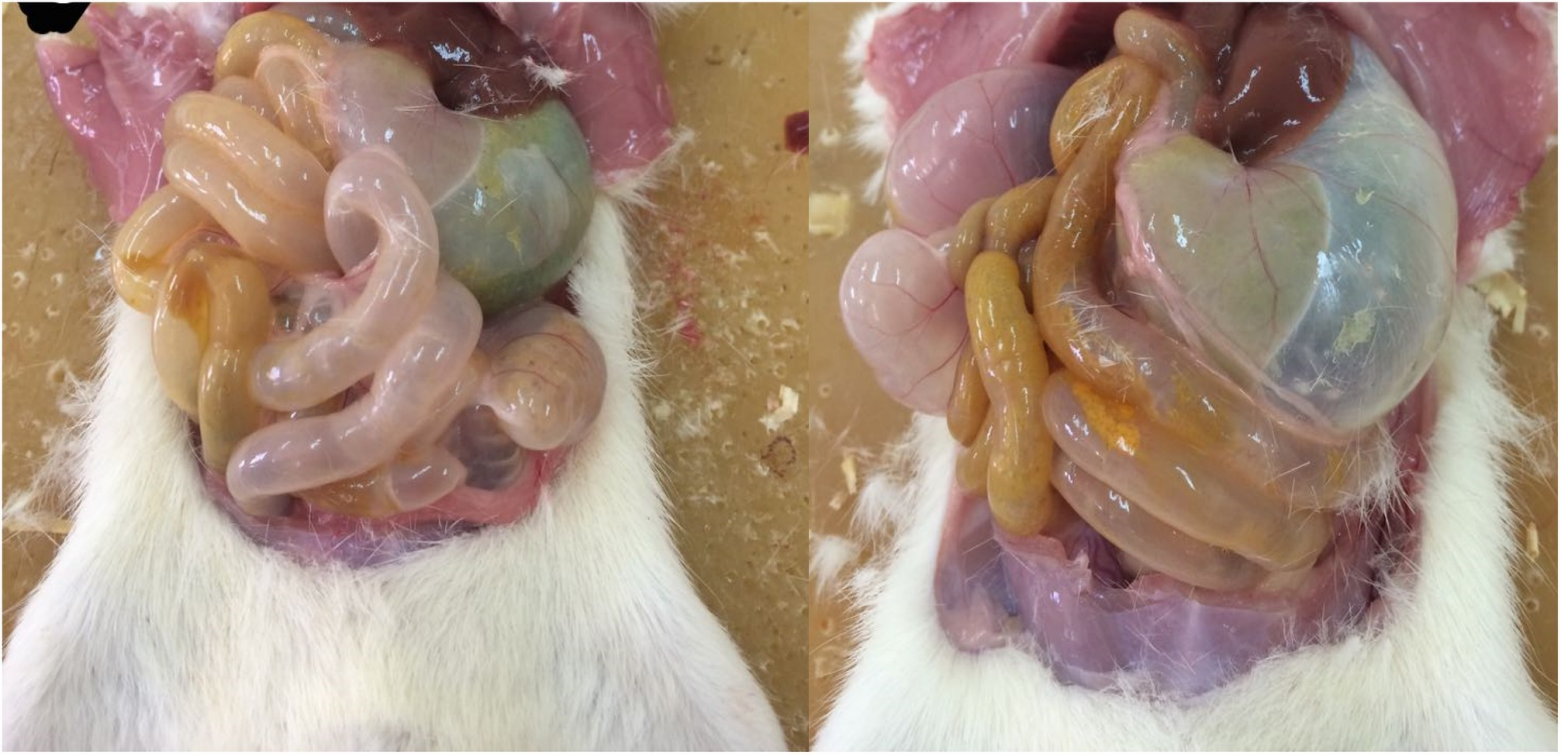
Two examples on the swelling of the gastro-intestinal tract of the rats belonging to the group of 500 mg aerogels.

The pilot study was designed to sight the highest safe dose of Ca-Alg aerogel when employed as a drug delivery system for orally administered short term treatments e.g. antibiotics. This was performed by monitoring the liver and kidneys enzymes. Four dosage levels were applied to specify the highest possible dosage of aerogels that can be used safely. One rat from the group of the highest dosage of 500 mg aerogels passed away on day 2 during the night, when no one of the staff member was available. At the end of the experiment, the remaining rats of the highest dose group were euthanized based on their behavioral signs. Upon necropsy examination of this group, the rats of this group were found to have swollen GI (Fig 2) that might be referred to a physical blockage of the gastro-intestinal tract because of the undigested Ca-Alg aerogel and the gases produced by its fermentation.

### ALP level analysis

**Fig 3:**
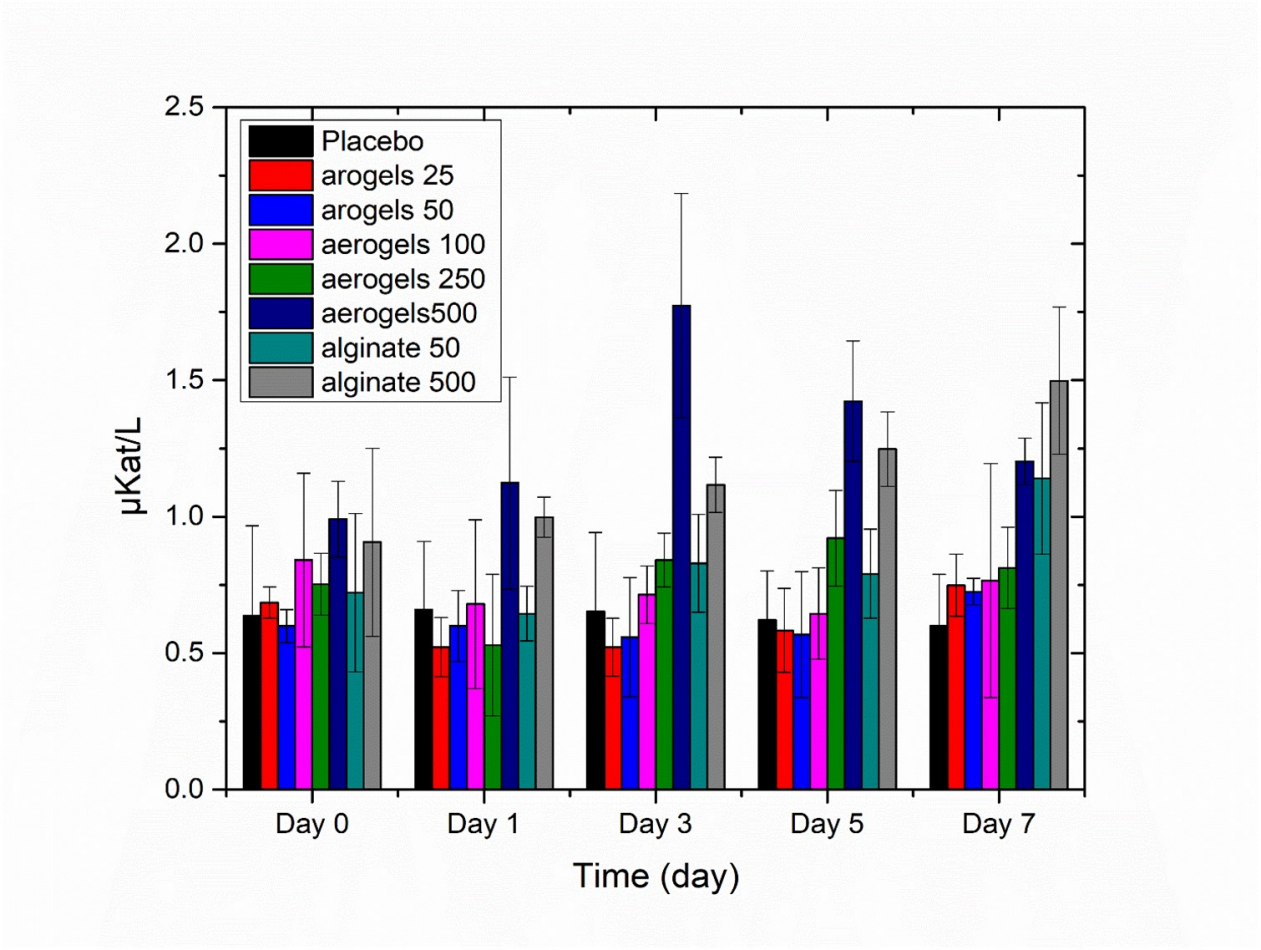
Short term analysis for the effect of different doses of calcium alginate aerogels and sodium alginate on ALP serum levels (aerogel corresponding to Ca-Alg aerogel and alginate corresponding to Na-Alg).

ALP blood level was used as an indicator for liver function. A baseline for the normal ALP level was established using the ALP readings for all the animals on day 0 (n = 34). The average of the established ALP level was 0.84 ± 0.36 μKat/L, and the median was 0.74 μKat/L, with a range of (0.28–1.98 μKat/L). ALP levels at five distinct time points (day 0, 1, 3, 5, and 7) are shown in Fig 3 for the seven treatment groups included in this part of the study. Overall, the average ALP levels for the different treatment groups of Ca-Alg aerogel and Na-Alg aerogel did not exceed neither the highest level of the normal range established at baseline nor the stated value in the literature (113.8 ± 4.7 IU/L = 1.93 μkat/L) [24]. It is well known however that the rise in the ALP values could be referred to stress factors that the rats might have experienced during blood sampling and /or during dose administration of test substances [25].

### Creatinine level analysis

**Fig 4:**
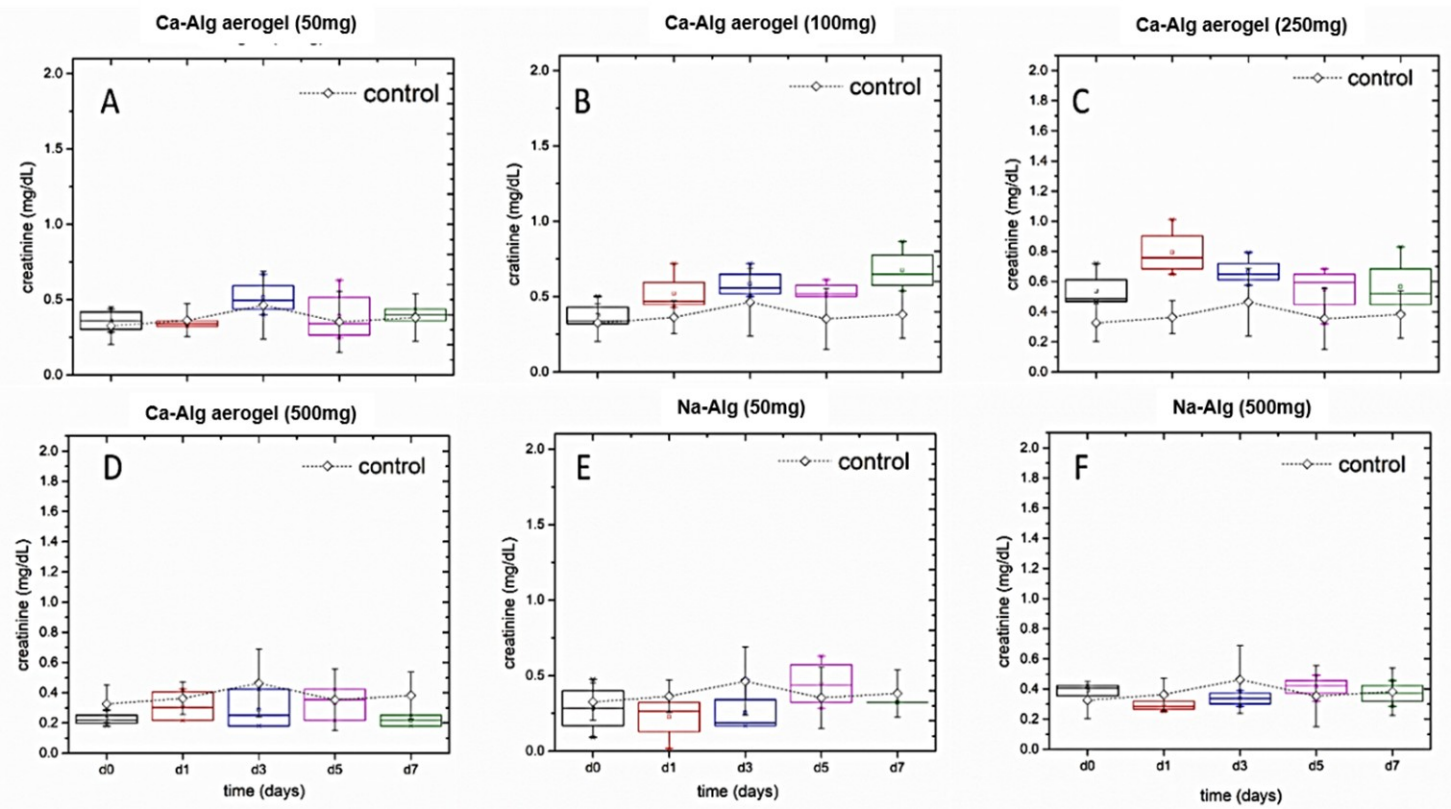
Box-plot for the in vivo serum creatinine level analysis that was done for the different doses of the aerogels and the alginate. The dotted lines in all the panels represent the creatinine values for all the control groups.

Creatinine blood level was used as an indicator for kidney function. The baseline for the normal creatinine level was also established using the readings for all the animals on day 0 (n = 34). The average of the established creatinine level was 0.38 ± 0.23 mg/dl, with a median of 0.32 mg/dl and a range of (0.09–1.48 mg/dl). When comparing the creatinine levels for the control group with the levels of each treatment group independently, creatinine levels of the 100 mg group (Fig 4B) increased slightly from 0.3 mg/dl at day 0 to 0.625 mg/dl at day 7. While in the group of 250 mg (Fig 4C) creatinine levels increased from 0.5 mg/dl to 0.72 mg/dl on day 1. Then, returned gradually to 0.5 mg/dl on day 7. With respect to the groups of rats treated with the following doses: 50 mg Ca-Alg aerogel (Fig 4A), 500 mg Ca-Alg aerogel (Fig 4D), 50 mg Na-Alg (Fig 4E) and 500 mg Na-Alg (Fig 4F), creatinine levels stayed within the range of 0.2 mg/dl to 0.5 mg/dl from day 0 to day 7. It was clear from the those results that the average of the creatinine level in all groups did not exceed neither the upper limit of the established baseline nor the normal level of creatinine in rats (0.25–3.09 mg/d) [26].

### Statistical analysis for the pilot study (one-way MANOVA)

The one-way multivariate analysis of variance (one-way MANOVA) was used to determine whether there are any differences between the seven groups when considered jointly on the ALP and/or creatinine levels. The multivariate result was statistically significant for the seven treatment groups, *F* (60, 102) = 2.18, *p* < .0005; Pillai’s Trace = 3.4, partial η^2^ = 0.562 indicating difference among the enzyme levels taken at different points of time. A separate ANOVA was conducted for each of the dependent variables, with each ANOVA evaluated at an alpha level of 0.025, and it showed a significant difference between groups on ALP and creatinine levels that were measured post treatment initiation. Most notably differences were detected in ALP on day3, *F* (6,21) = 5.1, *p* = .002; partial η2 = 0.59, day 5, *F* (6,21) = 5.72, *p* = .001; partial η^2^ = 0.62, and day 7 *F* (6,21) = 7.5, *p* < .0005; partial η^2^ = 0.68.

Creatinine levels started to show univariate differences from day 1, *F* (6,21) = 11.4, *p* < .001; partial η^2^ = 0.77, and it exhibited differences on day 3 *F* (6,21) = 5.9, *p* = .001; partial η^2^ = 0.63 and day 7 *F* (6,21) = 7.9, *p* < .0005; partial η^2^ = 0.69, but there was no significant differences on day 5, *F* (6,21) = 1.5, *p* = .23; partial η^2^ = 0.30.

Post hoc analysis using Tukey multiple comparison tests (Table SI1 in S1 File) further clarified the points of significant mean differences at an alpha level of 0.05. Where ALP levels on day 3 for the treatment group Ca-Alg aerogel at 500mg dose level was significantly different from all other treatment groups including the placebo but it was not different from Na-Alg at 500mg dose level. On day 5, ALP mean levels in the Ca-Alg aerogel-500mg and Na-Alg-500mg groups were extremely similar to each other (mean difference = 0.07, *p* = 1), and both did not exhibit differences to Ca-Alg aerogel-250 group but they were significantly different from the other remaining treatment groups. On day 7, Na Alg-500mg group demonstrated significant differences from all other groups except for Ca-Alg-aerogel-500 mg and Na Alg-50mg (p = 0.3 and 0.25 respectively).

As for creatinine mean levels, differences were seen starting from day 1 as shown in Fig 4C, where the treatment group of Ca- Alg aerogel- 250 was significantly higher than all other groups including control group (Table SI2 in S1 File), but these levels started to go down on subsequent days and differences from the control were not seen in any other instance, except for the Ca-Alg-aerogel-100mg on day 7 (*p* = 0.02).

The pilot study is limited due to the small sample size and individual variations among rats that is revealed by the high error bars in Fig 4. That being said, the increase in the creatinine levels (which is still within the normal ranges) observed in Ca-Alg aerogel groups can be explained by the presence of calcium in the aerogel. As it was reported by Barry et al. (2014) that daily calcium supplement causes a small increase in the blood creatinine, and the authors refer this increase to the effect of calcium on renal function, or to the mild vasoconstriction due to increased calciuria or induction of natriuresis by calcium, which can cause mild dehydration, or possibly increased calcification of the glomeruli [27, 28].

Based on the results obtained from the short term sighting study, including clinical observations and blood biochemistry, the dosage level of 250 mg aerogels (dose ~ 1000 mg/kg) was selected to be further investigated in the long term *in vivo* exposure study.

### Long-term *in vivo* exposure study (15 days repeated dose study)

#### ALP level analysis

**Fig 5:**
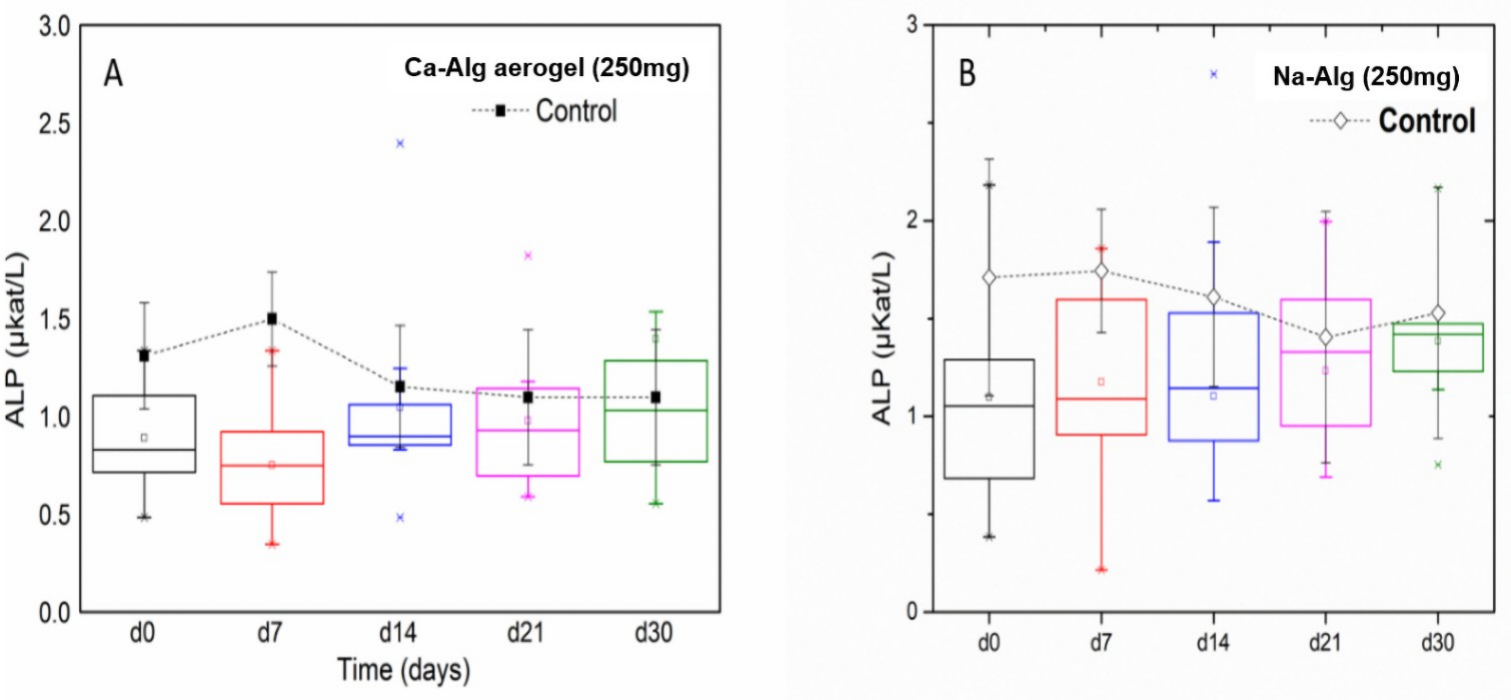
Box-plot for ALP test that was done for the 250 mg Ca-Alg aerogels (A) and Na-Alg (B) during a long-term experiment. The dotted lines in all the panels represent the ALP values for all the control groups.

In Fig 5A, the long-term experiment results for the ALP measurements of the Ca-Alg aerogel in comparison to the control group are illustrated. An independent-samples t-test was conducted to compare post-exposure initiation ALP levels (days 7 and 14) in the control group and the treatment group. There was not a significant difference in the levels for the control group (*M* = 1.50, *SD* = 0.24) and treatment group (*M* = 1.17, *SD* = 0.38) on day 7; *t*(14) = 1.9, *p* = 0.08 when equal variances are assumed. In addition, there was not a significant difference in the levels for the control group (*M* = 1.2, *SD* = 0.31) and treatment group (*M* = 1.15, *SD* = 0.31) on day 14; *t*(14) = 0.40, *p* = 0.70 when equal variances are assumed.

To compare ALP levels during recovery and after terminating the exposure to Ca-Alg aerogel (days 21 and 30), an independent-samples t-test was conducted in the control group and the satellite group, on both points of time. There was not a significant difference in the levels for the control group (*M* = 1.50, *SD* = 0.17) and satellite group (*M* = 1.26, *SD* = 0.47) on day 21; *t*(14) = 1.09, *p* = 0.30 when equal variances are assumed. In addition, there was a not a significant difference in the levels for the control group (*M* = 1.50, *SD* = 0.29) and satellite group (*M* = 1.85, *SD* = 1.70) on day 30; *t*(14) = -0.50, *p* = 0.63 when equal variances are assumed. Similarly, the average ALP level of the Na-Alg group (Fig 5B) were found to be within the normal ranges, though, it was noticed that the average level of ALP in the Na-Alg group was increasing gradually after cutting the treatment (day 14) till the end of the study time (but still within the normal levels), although the ALP level in the control of this group had an opposite trend.

An independent-samples t-test was also conducted to compare post-exposure and recovery ALP levels (on days 7 and 14, 21 and 30) between the control group and the Na-Alg treatment group. There was a not a significant difference at any of the time points. Thus, it is reasonable to infer that exposure to Ca-Alg aerogel or to Na-Alg has no short nor long- term impact on the hepatic function of Wistar rats when given at a daily dose of 250mg for a two weeks period. However, further investigation and longer experiments are needed to check the sustainability of this trend.

#### Creatinine level analysis

**Fig 6:**
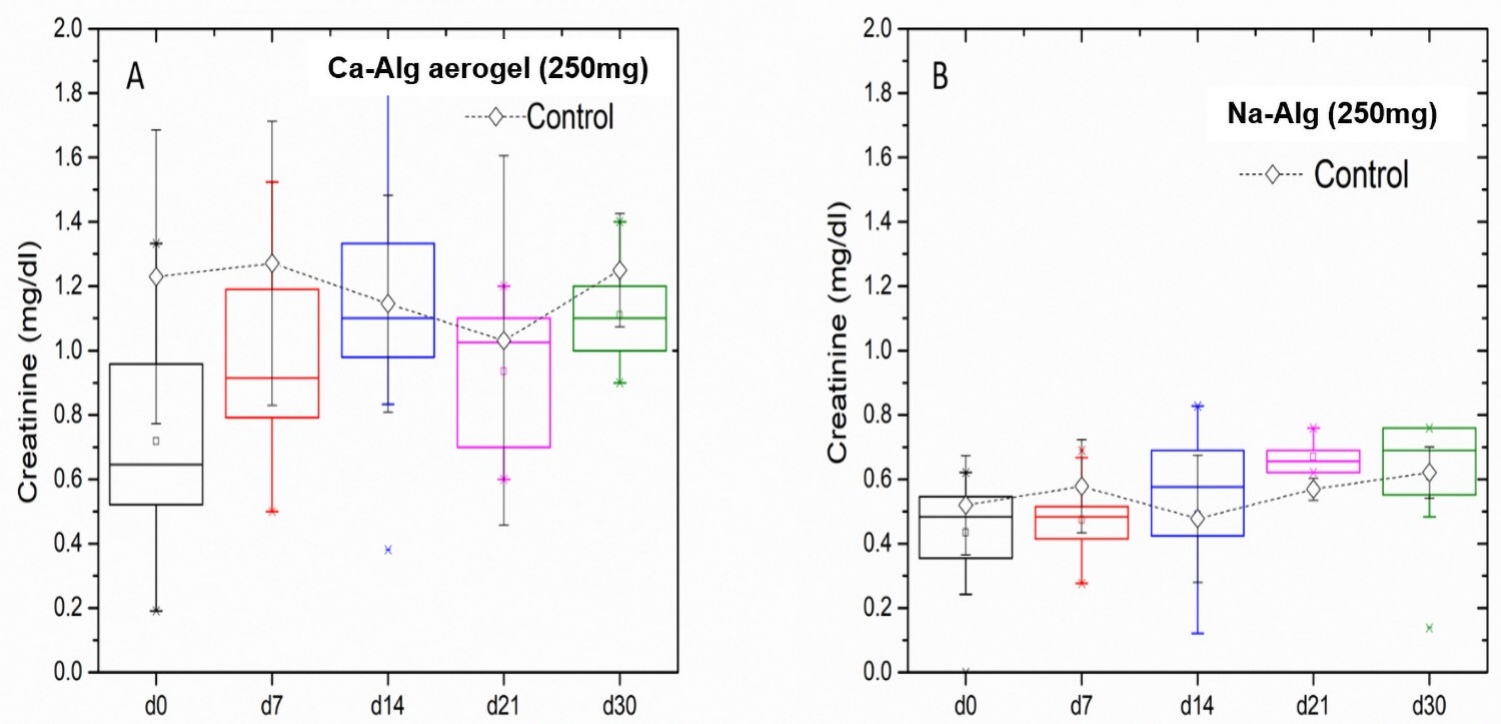
In vivo serum creatinine analysis, for the 250 mg Ca-Alg aerogels (A) and Na-Alg (B) during a long-term experiment.

In the long-term treatment group, creatinine values in the groups treated with Ca-Alg aerogel and Na-Alg was evaluated on day 0, and these values were used to establish the baselines for the readings. In long term treatment (Fig 6), both Ca-Alg aerogel and Na-Alg groups showed no toxic effect on the kidney, and the maximum serum creatinine mean level was observed in Ca-Alg aerogel group reaching 1.27 mg/dl at day 14.

An independent-samples t-test was conducted to compare post-exposure initiation creatinine levels (days 7 and 14) in the control group and the treatment group. There was not a significant difference in the levels for the control group (*M* = 1.27, *SD* = 0.44) and treatment group (*M* = 1.03, *SD* = 0.33) on day 7; *t*(14) = 1.25, *p* = 0.23 when equal variances are assumed. In addition, there was not a significant difference in the levels for the control group (*M* = 1.15, *SD* = 0.34) and treatment group (*M* = 1.35, *S*D = 0.25) on day 14; *t*(14) = -1.42, *p* = 0.18 when equal variances are assumed.

To compare creatinine levels after terminating the exposure to Ca-Alg aerogel (days 21 and 30 as recovery period), an independent-samples t-test was conducted in the control group and the satellite group, on both points of time. There was not a significant difference in the levels for the control group (*M* = 1.03, *SD* = 0.26) and satellite group (*M* = 0.94 SD = 0.23) on day 21; *t*(14) = 0.78, *p* = 0.45 when equal variances are assumed. In addition, there was not a significant difference in the levels for the control group (*M* = 1.25, *SD* = 0.08) and satellite group (*M* = 1.11, SD = 0.16) on day 30; *t*(14) = 1.97, *p* = 0.69 when equal variances are assumed.

An independent-samples t-test was also conducted to compare post-exposure and recovery creatinine levels (on days 7 and 14, 21 and 30) between the control group and the Na-Alg treatment group and between the control group and the satellite group respectively. There was not a significant difference at any of the time points.

The findings of this part of the study provide preliminary evidence that neither immediate nor delayed renal toxicity is recognizable when the Ca-Alg aerogels are given at a dose of 250 mg (approx. 1000 mg/KG) for 15 days. However, delayed effect on renal function cannot be totally ruled out in the case of Ca-Alg aerogel, and further laboratory evaluation is needed. The decrease of the mean creatinine level on day 21 can be interpreted as recovery when the exposure to Ca-Alg aerogel was stopped, specifically when comparing it to the rise that was observed on day 14.

### Shift in gut microbiota

Gut microbiota are active partners in the body health and gut metabolism of their host. The by-products of Alg digestion might result in products that inhibit colon cancer [29]. They are responsible for providing up to 15% of total caloric intake because they participate in polysaccharide digestion, as well as vitamins, short- fatty acids and other nutrients’ production for their hosts [30]. Therefore, it is important to study the effect of calcium alginate aerogel on the gut microbiota when assessing its safety.

**Fig 7:**
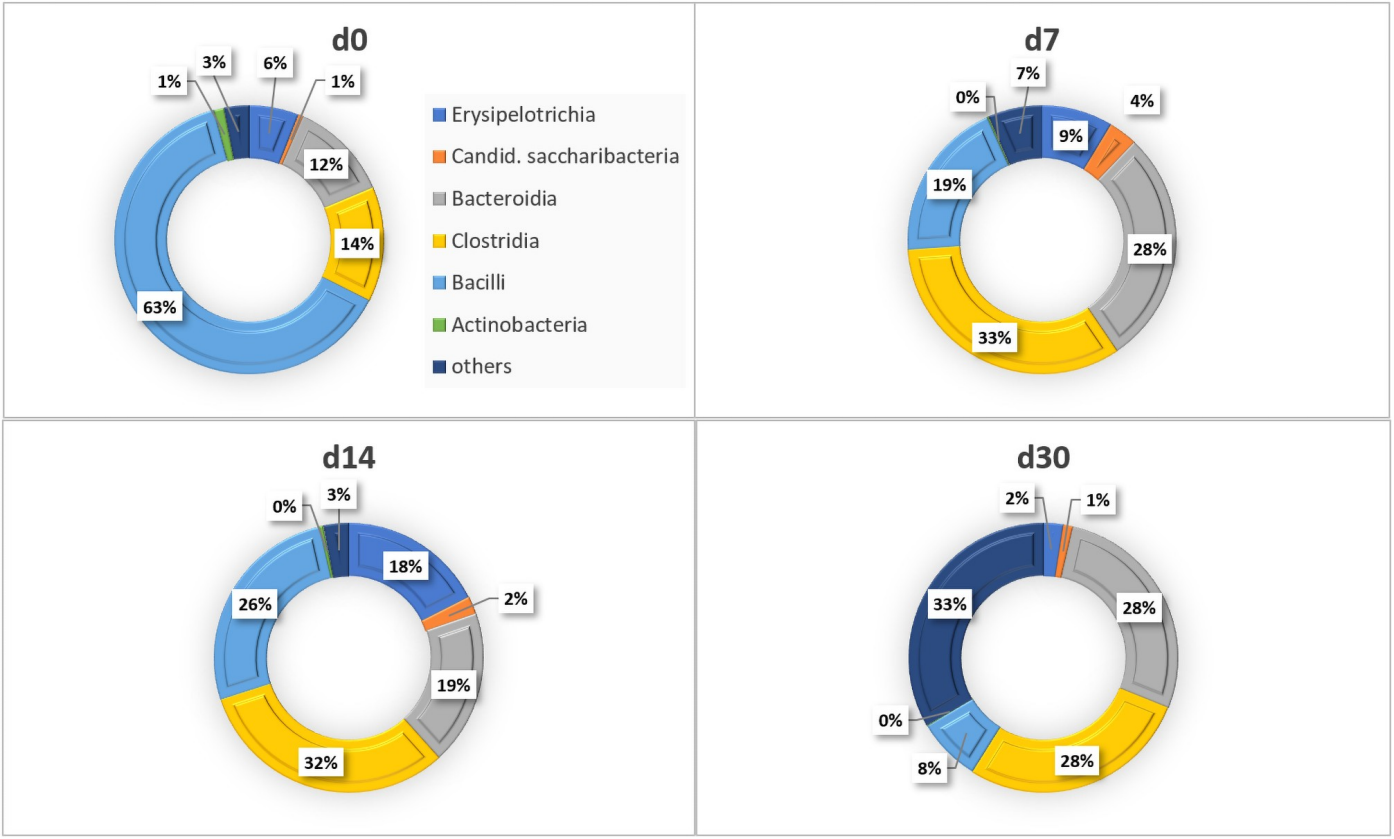
Microbial community structure of the gut at the class level in rats treated with Ca-Alg aerogel.

The remarkable results for the gut microbial study are summarized in Fig 7, which was obtained by analyzing the 16S rRNA gene sequencing. In the gut of the untreated rats and the rats before giving the Ca-Alg aerogel, the highest abundance of the bacterial members belonged to the class Bacilli, while members of the classes Clostridia, Bacteriodia, Erysipelotrichia, and Candidatus saccharibacteria were represented in lower relative abundances (control and rats at day 0, Fig 7). In response to feeding rats with Ca-Alg aerogel, gut microbiota showed different patterns; the relative abundance of certain groups of bacteria increased during the Ca-Alg aerogel regime and continued to increase after cutting it, such as Clostridia and Bacteriodia. While members of another groups (i.e., *Erysipelotrichia*, and *Candidatus saccharibacteria*) increased during Ca-Alg aerogel treatment and then decreased again at day 30. Members of the class Bacilli showed a unique trend that is after being the most abundant group at day 0 (63%), the relative abundance decreased dramatically until it reached < 5%, even after stopping the aerogel treatment.

**Fig 8:**
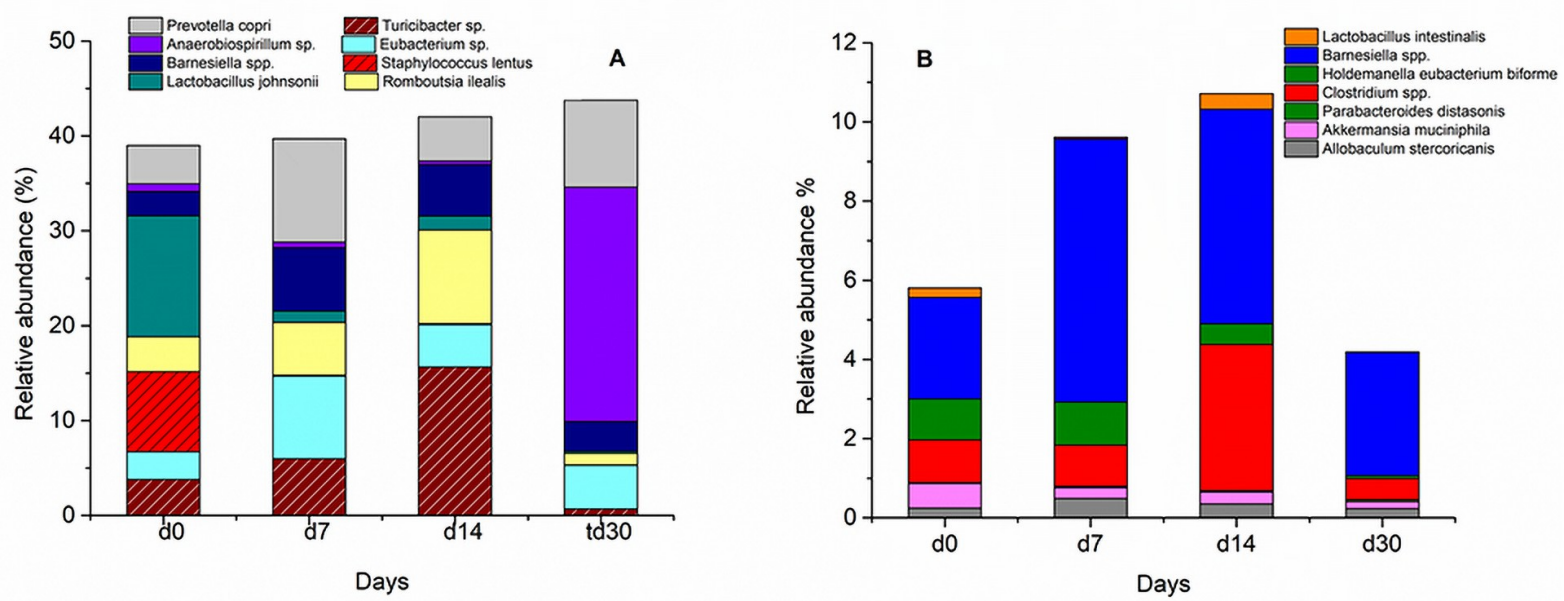
Temporal shift in gut microbiota in response to the Ca-Alg aerogel exposure, representing the species that increased, decreased, or totally disappeared due to the treatment.

On the species level (Fig 8A and 8B), gut microbiota showed similar response to that noticed at the class level, with either reversible or irreversible abundances at day 30 (after stopping Ca-Alg aerogel treatment). This response can be categorized into 5 distinctive trends based on the increase/decrease or disappearance of certain species in response to the treatment. The first one is when the relative abundance of the bacteria increased in response to aerogel treatment and then decreased back to the normal, or close to the normal, value. These include *Romboutsia ilealis*, *Turicibacter spp*., *Barnesiella spp*., *Allobaculum stercoricanis*, *Eubacterium spp*., *Clostridium spp*. and *Barnesiella spp*. This behavior may refer to the ability of such bacteria to digest calcium alginate aerogel, although to confirm this hypothesis, more experiments should be conducted. However, the current findings are in agreement with the previous results of Li et al. (2016), as they reported the capability of different species of Bacteroides to utilize alginate and its oligosaccharides [31].

The second trend (Fig 8A) is represented by some species, such as *Anaerobiospirillum*, that showed an irreversible massive increased abundance (from 1% at day 0 to 24% after stopping the aerogel). Such increase might induce diarrhea; it was reported in the literature that this kind of bacteria was isolated from patients suffering from diarrhea [32, 33]. Also, it was noticed when collecting the feces of rats treated with Ca-Alg aerogel that the feces was softer, lighter in color, and smaller in size compared to that of untreated rats, which might support such effect of raising the relative abundances of *Anaerobiospirillum*. It is also expected to have acetic acid and succinic acid as major byproducts of *Anaerobiospirillum*, as well as a trace of lactic acid [34], which in its role may alter the intestinal pH. Such alteration may have an impact on the interaction between the lactate-producing and lactate-consuming communities [35]. Moreover, Pereira and Gibson (2002) reported in their clinical study that lactic-acid-producing bacteria exert beneficial effects on host health, such as promoting cholesterol absorption [36]. Therefore, such pH alteration may have an effect on the cholesterol absorption in the host intestine. Another explanation for such irreversible massive increased abundance is increasing the fermentability with time. Brownlee et al. (2005) reported that the fermentability of Alg appear to be slightly lower in rats than it is in humans within 24 hours (64% in rats compared to 80% in humans). However, the fermentability in rats increases with continuous feeding of Alg (more than 4 weeks). Thus, feeding with alginate aerogel over time is expected to raise the number of the colonic microflora that ferment alginate [17].

The third trend that was observed in Fig 8A and 8B, is the decrease in bacterial growth with time during feeding with Ca-Alg aerogels and continue decreasing even after cutting it. Examples on these species are *Lactobacillus johnsonii* (acidophilus), *Akkermansia muciniphila* and *Holdemanella eubacterium biforme*. Such behavior might be attributed to the overgrowth of other types of bacteria, which were blooming during the period when the aerogels were abundant.

The fourth trend displayed total loss of the bacteria species after cutting the Ca-Alg aerogel treatment, this trend was related to some species such as *Staphylococcus lentus* and *lactobacillus intestinalis* (Fig 8A and 8B). However, the findings of the current study do not support the previous research finding by Wang et al. (2006), in which they reported that Alg oligosaccharide prepared through enzymatic hydrolysis of Na-Alg enhanced the growth of intestinal *bifidobacteria* and *lactobacilli* of male Wistar rats after feeding for two weeks [37]. Bereswill et al. (2017) investigated the potential of a murine commensal intestinal *L*. *johnsonii* (acidophilus) strain to reduce intestinal pathogenic burdens and to alleviate pro-inflammatory immune responses upon *C*. *jejuni* infection *in vivo* [38]. Bifidobacteria and lactobacilli are known to directly inhibit the growth of pathogenic bacteria, such as certain species of *Clostridia* (i.e., *Clostridium difficile* and *Clostridium perfringens*) and pathogenic Enterobacteriaceae, through the production of short-chain fatty acids, lowering of colonic pH, production of antimicrobial compounds, and competition for growth substrates and adhesion sites [39–41].

The last trend showed a decrease during the treatment with Ca-Alg aerogel and after stopping it there was a surprising blooming of the bacteria. This trend is shown in *Anaerobiospirillum sp*. and in *Prevotella copri* (Fig 8A). Such sudden increase in the relative abundances may be referred to certain byproducts resulted from the degradation of Ca-Alg aerogel, which might inhibit the growth of *Anaerobiospirillum sp*. and in *Prevotella copri*. Due to ceasing the production of these by-products (after cutting the Ca-Alg aerogel) these two species started to bloom again. Another assumption could be that due to the resulted by-products of Ca-Alg aerogel degradation. However, such assumptions need further studies to be confirmed.

Alginate oligosaccharides intervention changed the gut microbiota in mice towards the dominance of two phyla; Firmicutes and Bacteroides, and the Erysipelotrichaceae family was increased compared to the control [42]. Subsequently, microbial carbohydrate metabolism in the mice gut was significantly stimulated, while the microbial amino acid metabolism was inhibited [42]. *In vitro* investigation of the specific location in the gut, where the alginate is metabolized, emphasized that it can reach the colon unmodified because it was highly resistant to the enzymes in the upper gastrointestinal tract [31]. Thus, alginate serves as a substrate for the intestinal microbiota. Naturally, alginate is an acidic polysaccharide with a linear β (1→ 4)- linked glycuronan comprised of residues of β -D -mannosyluronic acid and its C-5 epimer α -l -gulosyluronic acid. These residues are arranged in a complex structure that can be homopolymeric or heteropolymeric [43]. Alginate degradation requires several steps, in which converting it into monosaccharides is a key step for its fermentation [44]. It can be degraded into smaller polymers or even monosaccharides by a group of alginate lyases that initiate the β-elimination reaction of the glucosidic bonds between the monosaccharide residues [45–47]. Microbial Alginate degradation results in the production of short chain fatty acids (SCFA), which provide a potential for alginate and its derivates to become a special food additive on human health [31].

## Conclusion

The aim of this study was to assess the effect of calcium-crosslinked alginate aerogel on the gut microbial community, and on the function of liver and kidney (through their enzymes, ALP and creatinine). The results revealed that neither immediate nor delayed renal or hepatic effects were identified when calcium-crosslinked alginate aerogel was given to the Wistar rats for 14 days with a daily dose of 250 mg. However, the renal impact and potential adverse effect cannot be entirely excluded in this case and further investigations are still warranted. Regarding the microbial community before and after the treatment with calcium-crosslinked alginate aerogel, gut microbiota showed different behaviors: Clostridia and Bacteriodia groups increased during the aerogels regime and continued to increase even after the aerogel was stopped, while other groups, such as Erysipelotrichia, and Candidatus saccharibacteria, increased during the aerogels treatment and then decreased again after one month. The blooming of some species appeared to have a suppression impact on others such as Bacilli, even after cutting the treatment with alginate aerogel. As a consequence of abundant increases of some bacteria, intestinal pH might be altered and such alteration may have an impact on the absorption of cholesterol. The increased abundance of some bacteria in response to the aerogel treatment and then the decreased abundant (back to the normal or close to the normal value) may refer to the ability of such bacteria to digest calcium alginate aerogel.

A limitation of this study was the fact that the number of animals was relatively small, and thus the results are expected to be improved with lower standard errors via an increased number of animals. It is needed to conduct this experiment for a longer time period in order to track the long-term effect of calcium-crosslinked alginate aerogel on the liver, kidney and gut microbial community. Nevertheless, the findings of this study enhance the understanding of the effect of alginate aerogel on the liver, kidney and gut microbial community. In combination with the literature finding, these results suggest that Ca-crosslinked alginate aerogel may be considered a safe potential drug delivery system for orally administered medicines.

## Supporting information

S1 File (PDF)
