# Supplementary material for: Evaluation of the orally administered calcium alginate aerogel on the changes of gut microbiota and hepatic and renal function of Wistar rats
Source: PLoS One. 2021 Apr 28;16(4):e0247633. doi: 10.1371/journal.pone.0247633 (PMC8081240; doi:10.1371/journal.pone.0247633)

Evaluation of the orally administered calcium alginate aerogel on the changes of gut microbiota and hepatic and renal function of Wistar rats

Mohammad A. A. Al-Najjar[¶*,1], Tamara Athamneh[¶*,2,3,#a], Reem AbuTayeh[1], Iman Bashiti[1], Claudia Leopold[3], Pavel Gurikov[4] and Irina Smirnova[2]

Table SI1. Tukey post hoc comparison of ALP mean levels during the experiment

Group 1	Group 2	MEAN DIFFERENCE	*STD ERROR*	p-Value
**Day 3**				
Ca-Alg-aerogel-500 mg	Control group	0.84*	0.20	0.01
	Ca-Alg-aerogel-50mg	0.95*	0.20	0.00
	Ca-Alg-aerogel 100mg	0.79*	0.20	0.01
	Ca-Alg-aerogel-250mg	0.67*	0.20	0.05
	Na Alg-50mg	0.74*	0.20	0.02
	Na Alg-500mg	0.40	0.20	0.49
**Day 5**				
Ca-Alg-aerogel-500mg	Control group	0.53*	0.15	0.03
	Ca-Alg-aerogel-50mg	0.51*	0.15	0.04
	Ca-Alg-aerogel-100mg	0.53*	0.15	0.03
	Ca-Alg-aerogel-250mg	0.25	0.15	0.65
	Na Alg -50 mg	0.44	0.15	0.11
	Na Alg -500mg	-0.07	0.15	1.00
Na Alg -500mg	Control group	0.60*	0.15	0.01
	Ca-Alg-aerogel-50mg	0.58*	0.15	0.01
	Ca-Alg-aerogel-100mg	0.60*	0.15	0.01
	Ca-Alg-aerogel-250mg	0.33	0.15	0.37
	Ca-Alg-aerogel-500mg	0.07	0.15	1.00
	Na Alg -50mg	0.51*	0.15	0.04
**Day 7**				
Na Alg -500mg	Control group	0.86*	0.16	0.00
	Ca-Alg-aerogel-50mg	0.77*	0.16	0.00
	Ca-Alg-aerogel-100mg	0.73*	0.16	0.00
	Ca-Alg-aerogel-250mg	0.69*	0.16	0.01
	Ca-Alg-aerogel-500mg	0.40	0.16	0.30
	Na Alg -50mg	0.40	0.16	0.25

Table SI2. Tukey post hoc comparison of creatinine mean levels during the experiment

**Group 1**	**Group 2**	**MEAN DIFFERENCE**	***STD ERROR***	**p-value**
		**Day 1**		
Ca-Alg-aerogel-250mg	Control group	0.43*	0.08	0.00
	Ca-Alg-aerogel-50mg	0.45*	0.08	0.00
	Ca-Alg-aerogel-100mg	0.27*	0.08	0.04
	Ca-Alg-aerogel-500mg	0.48*	0.08	0.00
	Na Alg -50mg	0.57*	0.08	0.00
	Na Alg -500mg	0.50*	0.08	0.00
Na Alg -50mg	Ca-Alg-aerogel-100mg	-0.29*	0.08	0.02
	Ca-Alg-aerogel-250mg	-0.57*	0.08	0.00
	Ca-Alg-aerogel-500mg	-0.08	0.08	0.94
		Day 3		
Ca-Alg-aerogel-250mg	Control group	0.20	0.09	0.32
	Ca-Alg-aerogel-50mg	0.15	0.09	0.64
	Ca-Alg-aerogel-100mg	0.08	0.09	0.97
	Ca-Alg-aerogel-500mg	0.38*	0.09	0.01
	Na Alg -50mg	0.41*	0.09	0.00
	Na Alg -500mg	0.33*	0.09	0.02
Na Alg -50mg	Control group	-0.21	0.09	0.30
	Ca-Alg-aerogel-50mg	-0.26	0.09	0.11
	Ca-Alg-aerogel-100mg	-0.33*	0.09	0.02
	Ca-Alg-aerogel-250mg	-0.41*	0.09	0.00
	Ca-Alg-aerogel-500mg	-0.03	0.09	1.00
	Na Alg -500mg	-0.08	0.09	0.97
		Day 7		
Ca-Alg-aerogel-100mg	Control group	0.29*	0.08	0.02
	Ca-Alg-aerogel-50mg	0.27*	0.08	0.03
	Ca-Alg-aerogel-250mg	0.10	0.08	0.84
	Ca-Alg-aerogel-500mg	0.45*	0.08	0.00
	Na Alg -50mg	0.35*	0.08	0.00
	Na Alg -500mg	0.30*	0.08	0.01
Ca-Alg-aerogel-250mg	Ca-Alg-aerogel-500mg	0.35*	0.08	0.00
Ca-Alg-aerogel-500mg	Ca-Alg-aerogel-100mg	-0.45*	0.08	0.00
	Ca-Alg-aerogel-250mg	-0.35*	0.08	0.00

INSTITUTIONAL REVIEW BOARD (IRB)

1/July/2019

Letter for IRB Protocol Approval

Approval Number: 2019-PHA-12

Research Title: Evaluation of the orally administered calcium alginate aerogel on the shift of gut microbiota and toxicity of Wistar rats.

Investigators: Mohammad A. A. Al-Najjar, Tamara Athamneh, Reem AbuTayeh, Iman Bashiti, Claudia Leopold, Pavel Gurikov, Irina Smirnova

Dear investigators,

The institutional review board in the faculty of pharmacy- Applied Science Private University has approved the above referenced protocol.

Date of approval: 1/July/2019, Date of expiration: 30/June/2020.

During this one year approval period, regular reviews are required. The protocol is due for a first review no later than 1/Oct/2019, and a second review no later than 1/Jan/2020.

The IRB staff will make every effort to send investigators regular reminders. However, investigators are responsible for submitting a regular review in advance of the review due date to ensure the continuity of IRB approval. It is very important that these deadlines are not missed. Failure to submit a regular review on time may result in the termination of the protocol. To continue this research beyond the one year approval period, a new protocol submission will be required.

It is the responsibility of the investigators to notify the IRB of any proposed changes regarding the work described within this protocol. Investigators agree that no such changes will be implemented until approval by the IRB, except where absolutely necessary to eliminate apparent immediate hazards to person(s).

Prof. Iman Basheti

IRB Chair

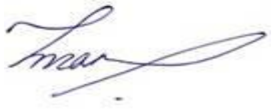

Supplement: S1 File — (PDF) [file pone.0247633.s001.pdf]
